# Antimicrobial Resistance in Pneumococcal Carriage Isolates from Children under 2 Years of Age in Rural Pakistan

**DOI:** 10.1128/Spectrum.01019-21

**Published:** 2021-12-22

**Authors:** Muhammad Imran Nisar, Shahira Shahid, Fyezah Jehan, Sheraz Ahmed, Sadia Shakoor, Furqan Kabir, Aneeta Hotwani, Sahrish Munir, Farah Khalid, Sajid Muhammad, Cynthia G. Whitney, Asad Ali, Anita K. M. Zaidi, Saad B. Omer, Najeeha Iqbal

**Affiliations:** a Department of Pediatric and Child Health, Aga Khan University, Karachi, Pakistan; b Department of Pathology and Laboratory Medicine, Aga Khan University, Karachi, Pakistan; c Emory University, Atlanta, Georgia, USA; d Bill & Melinda Gates Foundation, Seattle, Washington, USA; e Yale Institute for Global Health, New Haven, Connecticut, USA; Hartford Hospital

**Keywords:** PCV10, Pakistan, antimicrobial resistance, nasopharyngeal carriage

## Abstract

Antimicrobial resistance is an emerging public health concern. Ten-valent pneumococcal vaccine (PCV10) was introduced in Pakistan’s Expanded Program on Immunization (EPI) in 2012 as a 3 + 0 schedule without catchup. From 2014 to 2018, children <2 years were randomly selected in two rural union councils of Matiari, Pakistan. Nasopharyngeal swabs were collected using standard WHO guidelines by trained staff and processed at Infectious Disease Research Laboratory at The Aga Khan University, Karachi using culture on sheep blood agar and Multiplex PCR methods described by CDC, USA. Pneumococcal isolates were identified by optochin sensitivity and bile solubility tests. Isolates were then tested for antimicrobial susceptibility by standard Kirby-Bauer disk-diffusion method on Mueller-Hinton Agar (MHA) with 5% sheep blood agar as per Clinical & Laboratory Standards Institute (CLSI) recommendations. Of 3140 children enrolled, pneumococcal isolates were detected in 2370 (75%). Vaccine coverage improved from 41% to 68.4%. Out of the 2370 isolates, 88.4%, 37.6% and 25% were resistant to cotrimoxazole, tetracycline and erythromycin, respectively. There was no resistance to penicillin, ceftriaxone, and vancomycin. For erythromycin, resistance increased from 20% in 2014/15 to 30.8% in 2017/18 and for tetracycline it increased from 34.9% to 41.8% both of which were explained by an increase in prevalence of serotype 19A. Pneumococcal isolates were susceptible to penicillin, ceftriaxone, and vancomycin. They were largely resistant to cotrimoxazole and tetracycline. There was an increase in erythromycin and tetracycline resistance attributed to increasing prevalence of serotype 19A. Pneumococcal isolates from carriage and invasive disease should be closely monitored for antimicrobial susceptibility.

**IMPORTANCE** Antimicrobial resistance is an emerging public health concern particularly in low- and middle-income countries where there is poor regulation and easy availability of antibiotics. This is the first study from Pakistan to report antimicrobial resistance patterns of pneumococcus after vaccine introduction in the community. Pakistan was the first South-Asian country to introduce PCV10 in its Expanded Program on Immunization (EPI) in 2012 as a 3 + 0 schedule without catchup. In this study, we describe the PCV10 impact on antimicrobial resistance patterns of pneumococcal nasopharyngeal carriage in children younger than 2 years of age in a rural district in Pakistan after the introduction of the vaccine.

## INTRODUCTION

Antimicrobial resistance (AMR) is an emerging threat to global health particularly in low- and middle-income countries (LMICs) where there is poor regulation and easy over-the-counter availability of antibiotics (1). AMR rates are further exacerbated by a lack of antimicrobial stewardship and poor adherence to infection control guidelines (1). Pakistan has a huge burden of pneumococcal infections, with an estimated 14,400 pneumococcal-related deaths in children less than 5 years of age in 2015, despite the introduction of vaccines (2). For the reasons described above, a wide variety of antibiotics are freely prescribed for the management of these infections. This has led to the development of increasing resistance to empirical antibiotic regimens by pneumococci (3, 4). Increasingly, other classes of drugs like macrolides and fluoroquinolones are being frequently prescribed (5). Vaccines have been proposed as one of the tools in combating the epidemic of AMR by the virtue of reducing the infection reservoir in the community (6). Pneumococcal conjugate vaccines (PCVs), which were first introduced in the year 2000, have been highly successful in decreasing the burden of pneumococcal disease globally (7–9). Prior to PCV introduction, the highest penicillin and macrolide resistance was noted in pneumococcal serotypes more common in the pediatric population, i.e., serotypes 6B, 6A, 9V, 14, 15A, 19F, 19A, and 23F (10). Most of these serotypes were included in the PCV7 formulation; consequently, penicillin nonsusceptible invasive pneumococcal disease (IPD) rates declined significantly in the post-PCV7 period (10). However, the selective pressure offered by vaccination coupled with uncontrolled antibiotic consumption altered the carriage structure in the nasopharynx with nonvaccine serotypes (NVT) replacing vaccine serotypes (VT) and a dramatic rise in antibiotic nonsusceptible IPD (11). Serotype 19A has accounted for most of this rise (12).

Pakistan was the first country in South Asia to introduce the 10-valent PCV in its Expanded Program on Immunization (EPI) in early 2013 (13). It is given in a schedule of three doses at 6, 10, and 14 weeks of life and no catchup immunization (3 + 0 schedule) (13).

As part of our study to estimate the direct and indirect effect of PCV10 on children under 2 years of age in a rural population in Pakistan, we collected nasopharyngeal swabs from 3,140 children over a 4-year period of 2014 to 2018 (14). Here, we describe the change in the AMR patterns over time among pneumococcal isolates from these specimens.

## RESULTS

From 2014 to 18, nasopharyngeal swabs were collected from 3,140 children. A total of 2,370 pneumococcal isolates were positive for pneumococcus, revealing an overall colonization rate of 75% (14). PCV10 carriage declined from 16.1% in year 2014/15 to 9.6% in 2017/18 (*P value* for trend <0.001) as vaccination coverage increased from 41% to 68.4% (14).

### Antimicrobial susceptibility patterns.

**FIG 1:**
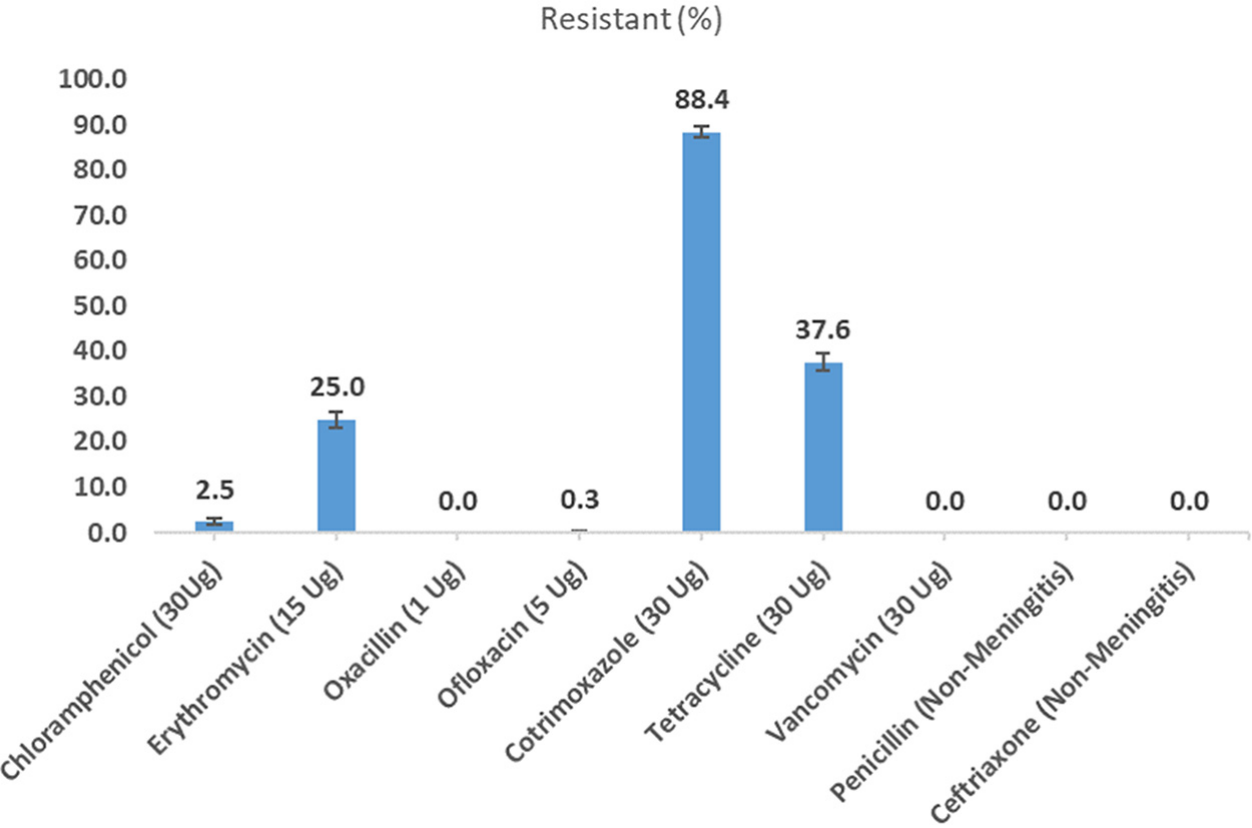
Percentage of pneumococcal carriage isolates (N = 2370) nonsusceptible by antimicrobial agent.

Out of the 2370 positive isolates, 88.4% (2094/2370, 95% CI 87 to 89.6%) were nonsusceptible to cotrimoxazole, 37.6% (891/2370, 95% CI 35.6 to 39.6%) were resistant to tetracycline, and 25% (592/2370, 95% CI 23.2–26.8%) were nonsusceptible to erythromycin. However, only 2.5% (59/2370, 95% CI 1.9–3.2%) and 0.3% (7/2370, 95% CI 0.1–0.6%) of the isolates were resistant to chloramphenicol and ofloxacin, respectively. None of the 2370 isolates were nonsusceptible to ceftriaxone, oxacillin, vancomycin, or penicillin (Fig. 1).

**FIG 2:**
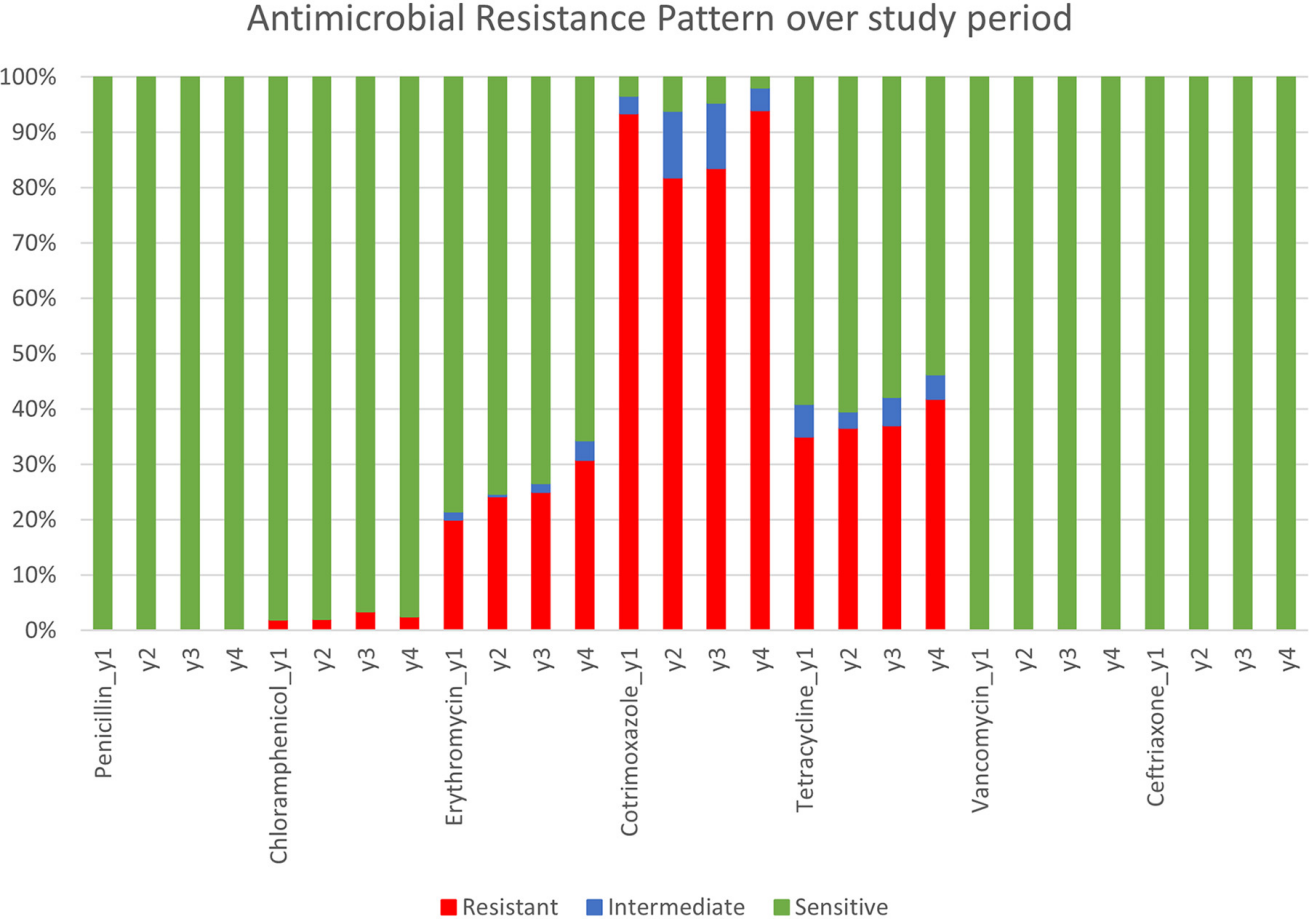
Percent of isolates susceptible to specific agents by year of the study.

The levels of nonsusceptibility to cotrimoxazole remained relatively constant throughout the 4 years of the study. Carriage of erythromycin-nonsusceptible pneumococci increased from 20% (125/623) to 30.8% (182/590) and for tetracycline it increased from 34.9% (218/623) to 41.8% (247/590). There were no other clear trends for changes in the prevalence of AMR patterns for other antibiotics (Fig. 2).

**FIG 3:**
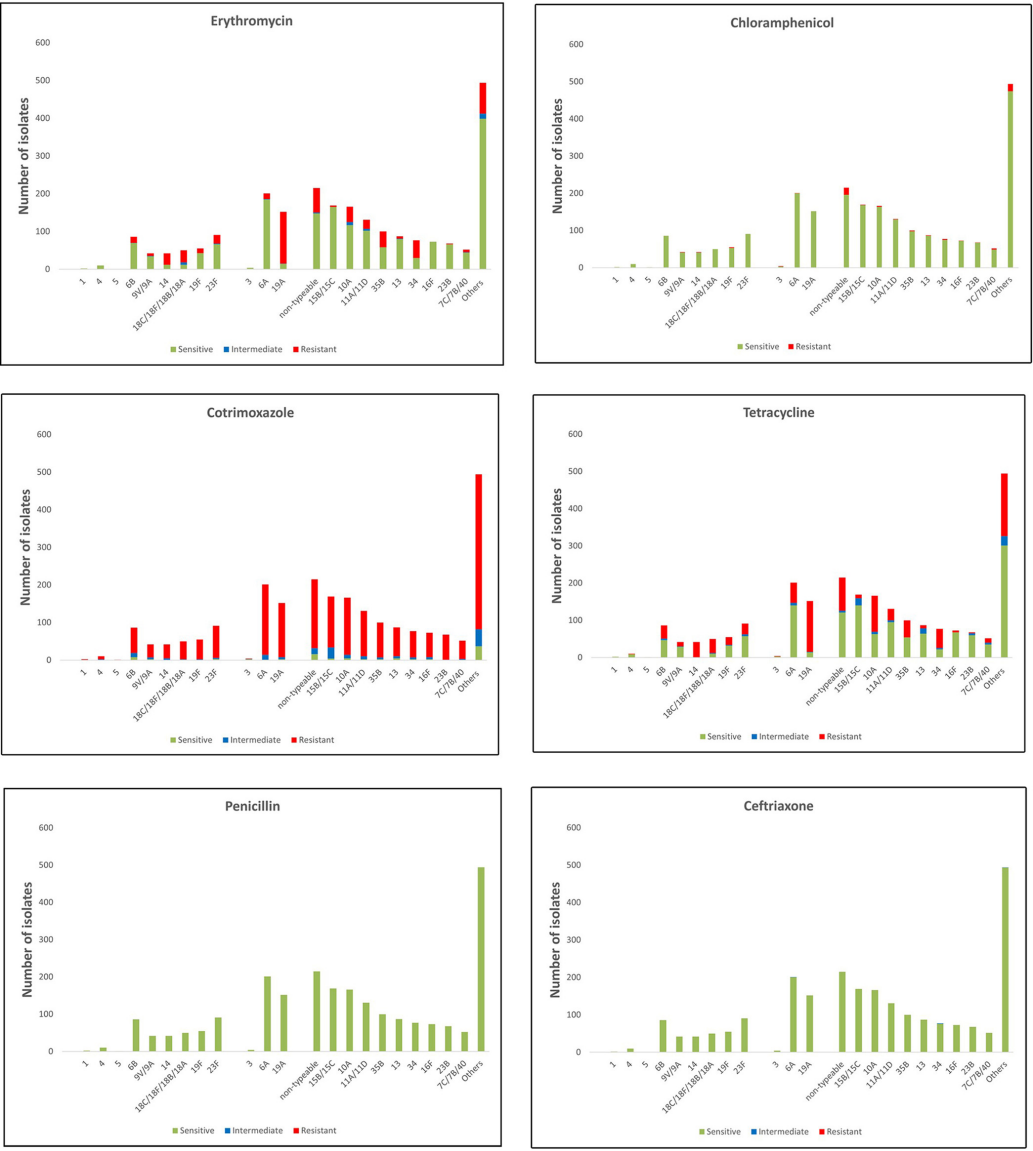
Serotype-specific susceptibility patterns of isolates.

NVT serotypes (*n* = 1989) were more prevalent than VT serotypes (*n* = 381) and they were more likely to be nonsusceptible to chloramphenicol (2.8% versus 1.0%, *P value* 0.049). VT serotypes were more likely to be nonsusceptible to erythromycin (31.5% versus 23.7%, *P value* 0.002) and tetracycline (46.7% versus 35.8%, *P value* <0.001) (Table 1). Most of the tetracycline nonsusceptibility was attributed to serotypes 19A (90.1%, 137/152 isolates), 8 (83%, 5/6 isolates), 10A (58.4%, 97/166 isolates), 34 (66%, 51/77 isolates), 18C/18F/18B/18A (76%, 38/50 isolates), and 14 (100%, 42/42 isolates). Most of the erythromycin nonsusceptibility was attributable to 19A (90.1% 137/152 isolates), 14 (71%, 30/42 isolates), 18C/18F/18B/18A (64%, 32/50), and 34 (61%, 47/77 isolates) (Fig. 3).

**TABLE 1:** Comparison of the proportions of VT and NVT pneumococcal carriage isolates susceptible, intermediate, or resistant to specific antimicrobial agents^*a*^

Antibiotic class	VT serotypes	NVT serotypes	*P* value
	N = 381 (%)	N = 1989 (%)	
Penicillin (nonmeningitis)			
Sensitive	381 (100.0)	1,989 (100.0)	
Chloramphenicol (30 μg)			0.049
Sensitive	377 (99.0)	1,934 (97.2)	
Resistant	4 (1.0)	55 (2.8)	
Erythromycin (15 μg)			0.002
Sensitive	251 (65.9)	1,485 (74.7)	
Intermediate	10 (2.6)	32 (1.6)	
Resistant	120 (31.5)	472 (23.7)	
Cotrimoxazole (30 μg)			0.95
Sensitive	14 (3.7)	80 (4.0)	
Intermediate	29 (7.6)	153 (7.7)	
Resistant	338 (88.7)	1,756 (88.3)	
Tetracycline (30 μg)			<0.001
Sensitive	189 (49.6)	1,180 (59.3)	
Intermediate	14 (3.6)	96 (4.8)	
Resistant	178 (46.7)	713 (35.8)	
Ceftriaxone (nonmeningitis)			0.750
Sensitive	381 (100.0)	1,986 (99.8)	
Intermediate	0 (0.0)	2 (0.1)	
Resistant	0 (0.0)	1 (0.1)	

a VT, vaccine type serotypes; NVT, nonvaccine type serotypes.

## DISCUSSION

We report the majority of the pneumococcal isolates to be nonsusceptible to cotrimoxazole (88.7%); one third of the isolates to be nonsusceptible to tetracycline (37.6%), and an increasing trend in the nonsusceptibility to erythromycin over the study period from 20.0% to 30.8%. We saw an increase in the prevalence of PCV13-specific serotype 19A, which was almost universally resistant to erythromycin and tetracycline. None of the isolates showed resistance to penicillin. This requires cautious interpretation. Though antibiotic resistance rates are rising in South Asia, major gaps exist in the AMR surveillance data for pneumococcus in the post-PCV period (15). In 2013, Shakoor et al. reported antibiotic susceptibility in 851 carriage isolates collected from children in lower income settlements in urban Karachi and rural Matiari. All (100%) of which were sensitive to penicillin (16). A recent study from Pakistan which described antimicrobial susceptibility rates in invasive pneumococcus also showed that all of the nonmeningeal isolates were sensitive to penicillin (17). In a systematic review of studies from 2004–2018 in Bangladesh, Ahmed et al. reported pneumococcal resistance rates for penicillin to be negligible (4%) (18). In Pakistan, antibiotics are easily available over the counter without prescription, which has resulted in their widespread nonjudicious use (19). In addition, many general practitioners and unlicensed health care providers prescribe broad spectrum antibiotics for mild, self-limiting illnesses (20, 21).This in parallel with poor compliance by patients results in increasing antibiotic resistance (5). Thus, we see widespread nonsusceptibility to cotrimoxazole, which was once the mainstay of treatment for community management of pneumonia (22, 23). We did not collect information on antibiotic prescription patterns as a part of our study; however, published literature from rural areas of Sindh report that 80% of the rural dwellers use antibiotics without prescription. The most sought-after antibiotics were amoxicillin (52.0%), tetracycline (16.9%), and cotrimoxazole (19). This widespread, over-the-counter use of amoxicillin threatens the current universal susceptibility of pneumococcus to this class of drugs. Our data indicate that using amoxicillin for pneumonia or ceftriaxone for meningitis, when warranted, remains good recommendations. Although the isolates are not from lung, blood, or CSF, they are not likely to be less resistant than invasive strains (and should be similarly resistant by serotype), so these data provide a quick check on whether treatment guidance remains appropriate (24).Cotrimoxazole has shown to be less effective in treating pneumonia as per WHO recommendations (25). Thus, its use for pneumonia or suspected pneumonia should be discontinued given resistance.

Previously, few studies in Pakistan have reported AMR patterns in pneumococcal isolates. Both studies were conducted in the pre-PCV10 period. In 1991, Mastro et al. collected invasive isolates from children with acute lower respiratory tract infection, 80% and 62% of blood isolates were nonsusceptible to tetracycline and cotrimoxazole, respectively, 39% isolates were resistant to chloramphenicol and no serotype was fully resistant to penicillin. More importantly, all isolates were susceptible to erythromycin, ceftriaxone, cefuroxime, and vancomycin (26). Our data suggest a change in the dominant nonsusceptible serotypes in Pakistan. In 1991, serotypes 19F, 16, 31, 5, and 6A were responsible for most of the resistance in disease whereas we found NVT serotypes to be mostly responsible in carriage isolates. Another baseline carriage survey conducted before PCV10 introduction reported 100% resistance to cotrimoxazole, 30% resistance to erythromycin, and 16.6% resistance in penicillin isolates. However, serotype-specific analysis was not performed in this study (27). A review of pneumococcal antimicrobial susceptibility trends in SAARC countries by Jaiswal et al. reported the mean pneumococcal IPD susceptibility rates against cotrimoxazole and erythromycin to decrease in the past 2 decades in South-Asia (28).

Post-PCV10 introduction, the pneumococcal carriage resistance rates for cotrimoxazole, tetracycline and erythromycin in this rural community were mostly higher than other PCV10 using countries. In Belgium, 1 year after the switch of vaccines from PCV13 to PCV10 and a coverage of around 89% for at least 2 doses, nonsusceptibility was most frequently seen for cotrimoxazole (34.3%), erythromycin (15.5%), and tetracycline (10.6%), and NVT serotypes 23B, 11A, 15A, 15B, and 35B dominated the nonsusceptible carriage isolates (29). In Brazil, 4 years post-PCV10 introduction and a vaccine coverage of 74.6% for three doses, the highest percentage of nonsusceptible carriage isolates was observed for cotrimoxazole (39.8%), erythromycin (28%), and tetracycline (26.3%). Serotype 19A was responsible for most of the erythromycin resistance. In contrast to our study, penicillin nonsusceptibility was seen in 39% of isolates (30). In Cyprus, PCV10 was introduced in a schedule of 2 + 1, the highest resistance rate was seen for erythromycin (27.5%) 4 years after introduction. The nonsusceptible rates were driven by NVT serotypes 23A and 15A. Serotype 19A emerged as a multidrug resistant strain and most of it was either intermediate or fully resistant to penicillin (31). In Kenya, HIV-infected children aged less than 5 years were observed 2 years after PCV10 introduction in a 3 + 0 schedule, more than 95% of pneumococcal isolates were nonsusceptible to cotrimoxazole, and approximately 80% were penicillin nonsusceptible; and the proportion was essentially unchanged during the study period. Penicillin intermediate susceptible carriage prevalence among PCV10 types declined significantly, but this decline was balanced by a significant increase in susceptible NVT carriage (32). Most of these settings show a high proportion of nonsusceptibility to cotrimoxazole, which may be due to increased use of this antibiotic for community acquired infections and in HIV-endemic settings. In Iceland 4 years after the introduction of PCV10 at a coverage of 98.7% for at least two doses, the highest resistance was seen for cotrimoxazole, tetracycline and erythromycin. Serotypes nonsusceptible to ≥3 antibiotic classes belonged to 15, 19F and 6C. Thus, NVTs, such as serogroup 15, 6C, 23B, and 19A, are emerging as resistant in the post-PCV10 vaccination era in many parts of the world (33).

In our study, the percentage susceptible of different serotypes against specific antibiotics varied little except for some serotypes like 19A, which was almost always nonsusceptible to erythromycin and tetracycline and contributed to the increase in their overall resistance. In other Asian countries, a high prevalence of erythromycin resistance was also observed in carriage isolates with the NVT serotypes 19A and 6A (86.0% and 85.7%, respectively) (34). In Japan, as demonstrated in the present study, serotype 19A emerged as a dominant serotype after PCV7 introduction, showing the highest rate of resistance genes among all the serotypes. Similarly, a high prevalence of these macrolide resistance genes in serotype 19A pneumococci was described in the United States, Europe, and Asia (Taiwan) (35–40).

In Matiari, the susceptibility rates changed little over the years except for erythromycin and tetracycline for which the resistance increased. Conversely, in Mozambique, 3 years post-PCV10 introduction in a 3 + 0 schedule with a coverage of 94.1% for 3 doses, a significant decline in proportions of isolates nonsusceptible to tetracycline (23.2% versus 14.4%) and erythromycin (13.8% versus 7.2%), in HIV-uninfected children and a reduction in proportion nonsusceptible to penicillin among pneumococcal isolates from HIV-infected children was noted over the study period. Proportions of nonsusceptible isolates for trimethoprim-sulfamethoxazole, penicillin, tetracycline, and erythromycin among both HIV-uninfected and HIV-infected children were highest for VT serotypes than NVT, in our study, NVT serotypes were more likely to be nonsusceptible (41).

We used the Kirby Bauer disc diffusion method for AMR testing. The method has been in use for more than 60 years now as it is relatively affordable and easy to perform. While the manual measurement of the inhibition zone may present a platform for inaccurate results, automated methods of measurement are now available and our test was standardized (CLSI guidelines) for testing pneumococci through use of specialized media, incubation conditions, and specific zone size interpretive criteria. In addition, we reported penicillin resistance only after confirmation with the penicillin MIC test.

## CONCLUSION

In summary, PCV13-specific serotype, 19A was responsible for the increase in erythromycin and tetracycline resistance. Fortunately, Pakistan has recently switched to PCV13 in its Expanded Program on Immunization. Out of the most prescribed antibiotics in the community, cotrimoxazole was largely resistant however there was no evidence of penicillin-nonsusceptible isolates following the introduction of PCV10, which is encouraging.

## MATERIALS AND METHODS

### Study setting and population.

This study was carried out between October 2014 and September 2018 in Khyber and Shah Alam Shah Jee Wasi, two rural union councils of Matiari, Sindh which have a total registered population of around 88,739. Approximately 15 children were enrolled every week after written informed consent from the guardians. Demographic and clinical information, vaccination history and a nasopharyngeal swab was collected from all enrolled participants (Tables S1 and S2). Samples were transported at 2–8^°^C and immediately stored upright at −80°C until further processing (42). For culture, batches of 20–40 samples were thawed, vortexed and 200 μl of the sample was added to a mixture of 1 ml rabbit serum, 5 ml Todd Hewitt broth with 0.5% yeast extract and incubated for 6 h at 37°C. After this, one loop full (10 μl) was inoculated onto bilayer sheep blood and colistin-nalidixic-acid-agar and streaked for isolation of streptococci. After 18–24 h, the plates were examined for the appearance of alpha-hemolytic colonies and susceptibility to optochin and bile solubility. DNA extraction was performed by crude boiling method; the resulting supernatant was collected in a sterile microcentrifuge tube and the remaining DNA extract was used for PCR techniques. Serotypes were deduced using conventional multiplex PCR assay and further confirmation was done by monoplex PCR (43, 44). The *cpsA* gene served as an internal positive control in all multiplex reactions. DNA of 2 μl was added to the PCR master mix containing nuclease free water, 2× Qiagen multiplex PCR buffer, Qiagen Q solution and 25 μM working stock of primers. Amplification was carried out in an Eppendorf Master Cycler Gradient with the specific temperature profile, and the amplified PCR products were stained and read under Bio-Rad Gel Doc imager. Serogroup 6 was additionally differentiated into serotypes 6A, 6B, 6C and 6D by same method used by Jin et al. with some modifications as mentioned earlier (45). Pneumococcal serotype controls added in each reaction were obtained from CDC streptococcal lab. The nontypeable products were confirmed by pneumococci using *lytA* real-time PCR per Carvalho et al. (46).

For quality control in the Optochin susceptibility and bile solubility reactions, Streptococcus pneumoniae ATCC 49619 and Enterococcus faecalis ATCC 29212 strains of American Type Culture Collection (ATCC) were used.

Detailed methods have been described elsewhere (14, 47).

### Antimicrobial susceptibility testing.

Isolates were tested for antimicrobial susceptibility by standard Kirby-Bauer disk-diffusion method on Mueller-Hinton Agar (MHA) with 5% sheep blood agar as per the Clinical & Laboratory Standards Institute (CLSI) recommendations (48). The antimicrobials tested were chloramphenicol (30 μg), erythromycin (15 μg), oxacillin (1 μg), ofloxacin (5 μg), cotrimoxazole (30 μg), tetracycline (30 μg), vancomycin (30 μg), penicillin (nonmeningitis), and ceftriaxone (nonmeningitis). (Table S3) Isolates of pneumococci with ≥ 20 mm zone diameter around oxacillin disk were considered susceptible to penicillin (MIC, MIC ≤ 0.06 μg/ml).

Isolates with reduced susceptibility to penicillin, i.e., ≤19 mm zone diameter around oxacillin disk were chosen to further determine both penicillin and ceftriaxone MICs. Penicillin was reported as resistant only after performing a penicillin MIC test. Macrolide resistance was tested by the ‘d-test’ in which two disks (erythromycin and clindamycin) were positioned 15 mm apart on an MHA plate followed by incubation of plates for 24 h at 37°C.

Streptococcus pneumoniae ATCC 49619 was used for Quality Control (QC) strains as recommended in CLSI 2012 (Table 2).

**TABLE 2:** Disk diffusion ranges for QC strain Streptococcus pneumoniae ATCC 49619 as per CLSI^*a*^

Antimicrobial agent	Disk content	Disk diffusion QC ranges, mm
Chloramphenicol	30 μg	23–27
Erythromycin	15 μg	25–30
Ofloxacin	5 μg	16–21
Oxacillin	1 μg	≤ 12
Penicillin	10 units	24–30
Tetracycline	30 μg	27–31
Trimethoprim-sulfamethoxazole	1.25/23.75 μg	20–28
Vancomycin	30 μg	20–27

a QC, quality control.

### Statistical analyses.

VT carriage was defined as isolation of any of the 10 serotypes included in PCV10 (serotypes 1, 4, 5, 6B, 7F, 9V, 14, 18C, 19F, 23F). NVT carriage was defined as presence of all other serotypes besides PCV10 specific serotypes, including nontypeable strains. PCV13 serotypes were defined as any of the serotypes included in the 13-valent PCV (1, 3, 4, 5, 6A, 6B, 7F, 9V, 14, 19A, 19F, 18C, and 23F). A study year ran from October of 1 year through September of next year. We described carriage rates by number of vaccine doses received and study year. We also described antimicrobial susceptibility patterns overall and by year of the study based on standard cutoffs described in Table S1, looking at trends for various VT and NVT serotypes individually and grouped together. *P value* of < 0.05 was considered statistically significant. All analyses were done using Stata version 15.0 and Microsoft Excel 2017.

Ethical approval was obtained from Aga Khan University’s Ethical Review Committee.

### Data availability.

The data set used to generate results in this article along with the codebook are available as online supplemental material.
