# Supplementary material for: Antimicrobial Resistance in Pneumococcal Carriage Isolates from Children under 2 Years of Age in Rural Pakistan
Source: Microbiol Spectr. 2021 Dec 22;9(3):e01019-21. doi: 10.1128/Spectrum.01019-21 (PMC8693922; doi:10.1128/Spectrum.01019-21)
Supplement: SUPPLEMENTAL FILE 3 — Supplemental material. Download SPECTRUM01019-21_Supp_1_seq7.pdf, PDF file, 0.1 MB [file spectrum01019-21_supp_1_seq7.pdf]

Table S1 Socio-demographic and clinical characteristics of enrolled children, n= 3140

	n (%)
**Age**	
Mean (SD) in months	10.5 (6.0)
0–3 months	304 (9.7)
4–11 months	1604 (51.1)
12-23 months	1232 (39.2)
**Gender**	
Male	1580 (50.3)
**Hospital Admissions in last year**	
None	3046 (97.0)
One	83 (2.6)
Greater than or equal to two	11 (0.4)
**Outpatient visits in last month**	
None	1611 (51.3)
One	797 (25.4)
Two	465 (14.8)
Greater than 2	267 (8.5)
**Smoker in household**	
Yes	1116 (35.5)
**Symptoms during last two weeks****	
Runny nose	1 584 (51.6)
Cough	1 220 (39.8)
Fever	1 463 (47.7)
Fast breathing	79 (2.6)
Difficulty in breathing	612 (19.9)
Lower chest indrawing	74 (2.4)
**Signs****	
Hypothermia <35^0 C	11 (0.4)
Hyperthermia >37.5	187 (6.1)
Tachypnea***	210 (6.9)
Lower chest indrawing	47 (1.5)
**Number of PCV10 doses received (Card verified/verbal)**	
3 doses	1809 (57.6)
2 doses	392 (12.5)
1 dose	352 (11.2)
0 dose	587 (18.7)

IQR= Interquartile Range, * defined as no. of persons/room, **data available for 3068 children, ***using WHO age-specific cutoffs

Table S2 Study enrollment details

	n
Number of households approached	4181

Number of children enrolled	3140
Number of nasopharyngeal swabs collected	3140
Number of samples positive for pneumococcus	2370

Table S3 *Streptococcus pneumoniae* antimicrobial susceptibility testing panel and zone diameter breakpoints as per CLSI

Zone diameter breakpoints as per CLSI (mm)				
**Antibiotic class**	**Abbreviation**	**Sensitive (S)**	**Intermediate (I)**	**Resistant (R)**
Chloramphenicol (30 µg)	C	≥ 21	NA	≤ 20
Erythromycin (15 µg)	E	≥ 21	16-20	≤ 15
Penicillin: Oxacillin (1µg)	OX	≥ 20	NA	NA
Ofloxacin (5 µg)	OFX	≥ 16	13-15	≤ 12
Cotrimoxazole (25 µg)	SXT	≥ 19	16-18	≤ 15
Tetracycline (30 µg)	TE	≥ 28	25-27	≤ 24
Vancomycin (30 µg)	VA	≥ 17	NA	NA
***Streptococcus pneumoniae* Minimal Inhibitory Concentration (MIC) (ug/ml)**				
Penicillin (non-meningitis)	P	≤ 2	4	≥ 8
Ceftriaxone (non-meningitis)	CRO	≤ 1	2	≥ 4
Vancomycin	VA	≤ 1	NA	NA
